# Real-Time Subject-Specific Predictive Modeling of PPG Signals for Artifact-Resilient SpO2 Estimation Under Hypoxia

**DOI:** 10.3390/s25237176

**Published:** 2025-11-24

**Authors:** Idoia Badiola, Swati Balaji, Diogo Silva, Vladimir Blazek, Steffen Leonhardt, Markus Lüken

**Affiliations:** 1 Medical Information Technology, Helmholtz Institute for Biomedical Engineering, RWTH Aachen University, 52074 Aachen, Germany; 2 Engineering Design Department, Indian Institute of Technology Madras (IIT Madras), Chennai 600 036, India; 3 The Czech Institute of Informatics, Robotics and Cybernetics, Czech Technical University in Prague (CTU), 166 29 Prague, Czech Republic

**Keywords:** photoplethysmography (PPG), predictive modeling, machine learning, peripheral arterial oxygen saturation (SpO2), hypoxia, artifacts, subject-specific modeling

## Abstract

Photoplethysmography (PPG) is widely used in health monitoring, but its reliability is often compromised by artifacts, limiting accurate peripheral arterial oxygen saturation (SpO2) estimation. Moreover, physiological and demographic factors can substantially alter PPG waveform morphology. We propose a lightweight, real-time predictive modeling approach that adapts to subject-specific PPG signal dynamics to improve monitoring robustness under conditions prone to artifacts. A total of 459 min of dual-wavelength PPG signals, together with reference SpO2 values, were collected from 17 healthy volunteers (2 female, 15 male, mean age 27±3 years old) undergoing controlled desaturation in the 85–100% range after being instructed to remain still. Cardiac pulses were segmented and decomposed into AC and DC components, and the adequacy of several signal models, ranging from sums of Gaussians to Fourier series, and polynomial expansions of different orders, was evaluated. A space of representative signal features was built from the best-performing model, and used to generate machine learning-based predictions for each pulse using the preceding four clean pulses. Predicted pulses could be directly compared with their originals, enabling accurate error estimation without simulated data. The predicted signals closely matched the originals, achieving mean $R^{2}$ scores above 0.9, and an SpO2 estimation RMSE of 1.28%. In practical use, the same approach could be applied to overcome artifact-corrupted segments if combined with a signal quality assessment module. Therefore, this algorithm provides a promising pathway toward more reliable SpO2 monitoring in wearable systems, particularly under hypoxic conditions.

## 1. Introduction

Photoplethysmography (PPG) is a widely used non-invasive, cost-effective optical technique for monitoring vital signs, including heart rate and peripheral oxygen saturation (SpO2) [1]. Its simplicity and affordability make it ideal for integration into wearable devices, enabling continuous health monitoring in both clinical and consumer settings. The principle of PPG is based on detecting changes in light absorption by tissues, which vary according to blood volume changes caused by the cardiac cycle. The signal comprises two main components: the alternating current (AC) component, reflecting pulsatile blood flow, and the direct current (DC) component, representing static tissue and non-pulsatile blood. These dual components enable PPG to provide a wealth of physiological insights, including heart rate, respiratory rate, and SpO2 [1,2,3].

SpO2 is primarily calculated through pulse oximetry, which uses two wavelengths, typically red (660 nm) and near-infrared (940 nm), to distinguish between oxyhemoglobin and deoxyhemoglobin. These measurements are combined using the ratio-of-modulation (R-value or *R*), a critical intermediate metric for SpO2 estimation [3,4], defined by the relative amplitudes of the AC and DC components at the two wavelengths:

(1) $$R=\frac{\left({\mathrm{AC}}_{\mathrm{RED}}/{\mathrm{DC}}_{\mathrm{RED}}\right)}{\left({\mathrm{AC}}_{\mathrm{IR}}/{\mathrm{DC}}_{\mathrm{IR}}\right)}.$$

SpO2 ($SpO_{2}$) is then estimated by applying a calibration curve—usually a linear regression—specific to the sensor and device configuration [5]:

(2) $$SpO_{2}=a\cdot R+b.$$

The calibration curve is generally obtained by linearly relating the R-value from the PPG measurements, *R*, with the clinical gold standard for arterial oxygen saturation (SaO2)—blood gas analysis (BGA) via arterial sampling and co-oximetry—with data from a desaturation study on several subjects.

Although the FDA recommends a root mean squared error (RMSE) with 95% confidence intervals (CI) below 3% in pulse oximeters compared to BGA values [6,7], most medical-grade devices typically claim an error of less than ±2% [8].

However, while PPG holds immense potential for real-time monitoring, its susceptibility to motion artifacts (MAs) remains a significant challenge. MAs are distortions caused by motion, sensor displacement, or poor skin contact, which introduce irregularities such as baseline wandering and spikes. These distortions affect the AC and DC components, leading to inaccurate parameter estimation [9,10].

MA removal is essential for the reliable use of PPG in both clinical diagnostics and wearable applications. Numerous strategies have been proposed to mitigate MAs, including reference-based and reference-free methods. Reference-based methods, including those utilizing accelerometer signals, have demonstrated significant potential. For example, Peng et al. [9] used constrained independent component analysis (ICA) combined with adaptive filtering, while Wu et al. proposed a DC removal technique with a recurrent least-squares adaptive filter [11]. Although effective, such methods depend on auxiliary sensors, increasing system complexity and cost.

To avoid reliance on external references, reference-free methods such as statistical filtering or synthetic reference generation have been explored. For instance, Kim and Yoo also employed a combination of ICA and block interleaving with low-pass filtering using PPG measurements at two different wavelengths [12] and Hanyu et al. used statistical indices like skewness and kurtosis to detect and remove corrupted PPG segments [13], whereas Sadhukhan et al. removed the noise frequencies by suppressing the corresponding Discrete Fourier Transform coefficients [14]. Reddy and Kumar applied singular value decomposition [15], and, similarly, Raghuram et al. employed complex empirical mode decomposition to generate synthetic references [16]. While these methods are less hardware-dependent, they often result in incomplete signal reconstruction or the loss of critical information. Furthermore, none of these algorithms were further validated for SpO2 estimation below normoxia levels after the motion artifact removal.

Recent advances in machine learning have also been applied to MA removal. Cycle-GAN-based methods [17] and other neural network approaches [18] have shown promise in denoising PPG signals. For instance, Afandizadeh Zargari et al. [17] employed generative adversarial networks to reduce noise, achieving significant improvements in signal quality metrics. Similarly, Shuzan et al. [19] proposed a method for estimating respiratory rate and SpO2 using machine learning models that integrate PPG signal features with variational mode decomposition to mitigate motion artifacts. However, these methods are often computationally expensive and require relatively long input segments, which limits their suitability for lightweight, real-time applications.

Parallel to artifact removal, efforts to model PPG waveforms have yielded methods to parameterize the signal for improved understanding and processing. Sološenko et al. used Gaussian and gamma functions to model PPG signals [20], while Martin-Martinez et al. predicted waveform evolution using autoregressive models [21]. Furthermore, Fourier series and polynomial functions—both recognized as reasonable function approximators when of sufficiently high order—were also employed due to their inherent unidimensionality, with the order serving as their sole degree of freedom. Harmonic modeling via Fourier series has been shown to approximate PPG wave-shape morphology effectively while maintaining computational simplicity [22,23]. Low-order polynomial functions have also been used to approximate individual PPG pulses or short segments, providing smooth, compact representations of the AC component that capture key morphological features such as the systolic upstroke and dicrotic notch [24]. These techniques demonstrated good performance in reconstructing PPG signals, but most of them were not directly applied to MA correction and none for SpO2 estimation.

Despite the advancements and related works to mitigate MAs [9,11,13,16,17,18,20,21], challenges remain in developing computationally efficient, accurate, and generalizable methods for PPG-based SpO2 estimation in the presence of MAs, as most studies assessing the efficacy of artifact removal by evaluating the accuracy of heart rate and respiratory rate estimations from reconstructed signals [10,25,26,27] are computationally too expensive for real-time applications, discard artifact-corrupted signal segments [28,29,30], or fail to validate the estimation of SpO2 under hypoxic situations [9,17,31,32,33,34,35,36].

Some other algorithms, such as altering the frame length of Molgedey and Schuster ICA by Fan et al. [37] and the variable mode decomposition by Tang et al. [38] were only tested on simulated data or on a single subject [39], which might be an over-simplification of the problem, especially when dealing with SpO2 estimation, as intrinsic physiological and demographic factors, such as skin pigmentation, sex, age, and vascular properties, can substantially alter the PPG waveform morphology and its optical absorption characteristics [40,41,42,43,44,45,46,47]. This variability also underscores the need for adaptive algorithms that can account for inter- and intra-subject differences. In particular, for machine learning-based approaches, it is critical that models do not merely generalize across heterogeneous training datasets but also preserve subject-specific features, thereby enabling accurate monitoring that respects individual physiological particularities [48,49].

This work seeks to address these challenges by building on state-of-the-art PPG modeling and MA removal techniques. By focusing on lightweight, patient-specific methodologies, this study aims to pave the way for more reliable PPG reconstruction techniques that allow for accurate SpO2 estimation in dynamic, real-world environments.

## 2. Materials and Methods

### 2.1. Experimental Study

Data were collected from a study involving 17 young, healthy subjects (mean age: 27±3 years old, 2 female and 15 male) with skin tones ranging from type I to type V on the Fitzpatrick Skin Phototype Classification scale (FSPC) [50], all of whom had given informed consent. The study was conducted according to the ethical standards presented in the 2013 Declaration of Helsinki, and subjects underwent a cardiac stress test under the supervision of a qualified physician to ensure their suitability for participation.

Unfiltered PPG signals for red (660 nm) and infrared (940 nm) wavelengths were recorded from a finger-clip sensor attached to the right index finger in transmissive configuration using Vasoport (ELCAT GmbH, Wolfratshausen, Germany) at 100 Hz. The patient monitor Intellivue MX700 (Philips Healthcare, Amsterdam, The Netherlands) was employed to record reference heart rate and SpO2 data. Subjects were connected to the CellAirOne hypoxia therapy device (TUR Therapietechnik GmbH, Rostock, Germany) through a breathing mask (TUR Therapietechnik GmbH, Rostock, Germany) to induce desaturation events in SpO2, simulating conditions similar to those experienced during certain medical procedures.

Each subject underwent three stages of 4 min of hypoxia (SpO2 down to 83–86%) and 5 min of hyperoxia (SpO2 above 99%). Each measurement lasted 27 min, during which participants were instructed to remain sitting still with their hands placed on their lap, resulting in a total of 459 min for the entire dataset. Data from all devices were synchronized and saved for further analysis.

### 2.2. Methods Overview

**Figure 1:**
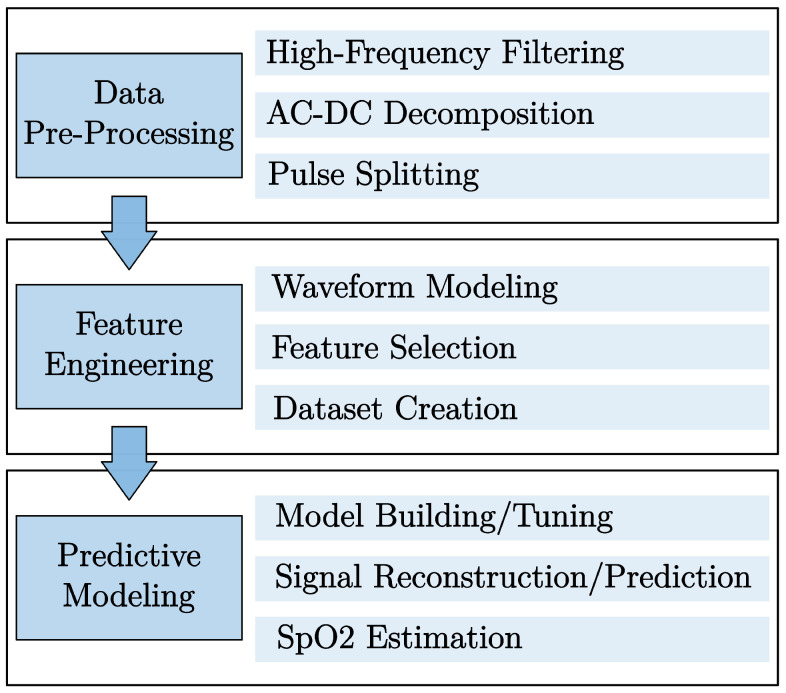
Overview of the implemented approach.

Figure 1 shows a summary of the methods employed in this work, which were implemented using MATLAB R2019a (Mathworks, Natick, MA, USA) and Python (Python Software Foundation, Wilmington, DE, USA, version 3.7).

The workflow consists of a first stage of pre-processing, where AC and DC components of the PPG signals from the red and the infrared channels were extracted from the PPG signal, and each cardiac pulse was identified.

The second stage involved feature engineering, which included waveform modeling of the DC component with a linear regression, and modeling of the AC component using sums of Gaussians [20], Fourier series expansions [22,23], and polynomial expansions [24] of several orders. The best fit was selected based on the adjusted coefficient of determination ($R_{\mathrm{adj}}^{2}$), which is a corrected goodness-of-fit (model accuracy) measure that penalizes the models for adding unnecessary features.

The last stage consisted of predictive modeling and evaluation of the accuracy of the forecasted pulses to estimate the SpO2 accurately. For that, the dataset was divided into train, validation, and test datasets, and extreme gradient boost (XGBoost) and ridge models were employed to find the best parameters for the best-performing model to predict the upcoming pulse based on the previous four cardiac pulses.

The performance of the proposed models was evaluated in two dimensions: (i) the reconstruction accuracy of the PPG waveforms and (ii) the accuracy of arterial oxygen saturation (SpO2) estimates derived from the model-predicted signals. The quality of pulse reconstruction was quantified using the coefficient of determination ($R^{2}$) for the pulsatile (AC) component and the mean absolute percentage error (MAPE) for the baseline (DC) component.

The accuracy of SpO2 estimation was assessed in two stages. First, the R-values were computed from both the original and predicted pulses (see Equation (1)), and their agreement was analyzed using Bland–Altman plots, with the 95% limits of agreement (LoA) reported. Second, calibration curves were derived for both signal sets by relating the corresponding R-values to the reference SpO2 measurements obtained from the pulse oximeter (see Equation (2)). This step enabled propagation of the LoA through the calibration relationship to determine the LoA in SpO2. Additionally, the root mean squared error (RMSE) between the reference SpO2 values and those estimated from the model-predicted pulses was computed to quantify overall prediction accuracy.

The detailed procedures for each of these analyses are presented in the following subsections.

For clarity, the terms “reconstructed,” “predicted,” and “forecasted” are used interchangeably throughout this text.

### 2.3. Data Pre-Processing

The red and infrared channels of the PPG signals were filtered using an equiripple finite impulse response (FIR) fourth-order filter to remove high-frequency artifacts below 10 Hz and separated into AC and DC components using fourth-order Chebyshev II infinite impulse response (IIR) filters—a band-pass filter between 0.67 and 4.5 Hz for AC and a low-pass filter below 0.67 Hz for DC [51].

The filtered PPG signal components were split into pulses using the open-source MATLAB package ppg-beats [52], based on the algorithm heartpy [53]. The split was made on the PPG obtained from the red channel, and the data were split synchronously for the IR channel.

Although participants were instructed to minimize movement during the 27-min recording period, slight involuntary movements or variations in finger-clip pressure may still have introduced motion artifacts. Importantly, rather than excluding these segments, all pulses were retained in the analysis. By retaining all pulses, including corrupted segments, the evaluation directly reflects the conditions under which prediction algorithms are expected to operate in practice. This design choice makes it possible to characterize not only the algorithm’s performance using perfectly clean signals, but also its behavior when predictions are attempted from non-ideal inputs or pulses. It is anticipated that incorporating established artifact detection methods to exclude corrupted pulses from the pool of reference inputs used for reconstructing the next target pulse would further improve performance.

### 2.4. Feature Engineering

#### 2.4.1. Waveform Modeling and Feature Selection

The PPG signal has a distinct semi-periodic waveform that can be modeled as the superposition of three additive components—a cardio-synchronously oscillating AC, a quasi-static DC component dominant in amplitude, and noise ϵ—yielding the model

(3) $$\begin{array}{cc} y^{k} & =y_{\mathrm{AC}}^{k}+y_{\mathrm{DC}}^{k}+\epsilon ,\\ \; & \approx f_{\mathrm{AC}}\left(t|\theta_{\mathrm{AC}}^{k}\right)+f_{\mathrm{DC}}\left(t|\theta_{\mathrm{DC}}^{k}\right)+\epsilon , \end{array}$$

where $y^{k}$ can interchangeably denote the PPG signal of the *k*-th pulse from the red or IR channels. In Equation (3), $f_{\mathrm{AC}}\left(t|\theta_{\mathrm{AC}}^{k}\right))$ is the functional parametrized by $\theta_{\mathrm{AC}}$, and $f_{\mathrm{DC}}\left(t|\theta_{\mathrm{DC}}^{k}\right))$ is the linear functional parametrized by $\theta_{\mathrm{DC}}\in R^{2\times 1}$:

(4) $$f_{\mathrm{DC}}\left(t|\theta_{\mathrm{DC}}^{k}\right)=\theta_{\mathrm{DC},0}^{k}+\theta_{\mathrm{DC},1}^{k}\cdot t.$$

Feature extraction refers to the process of solving the optimization problem

(5) $$\theta^{k}=\underset{\theta^{k}}{\mathrm{argmin}}{∥y^{k}-\left(f_{\mathrm{AC}}\left(t|\theta_{\mathrm{AC}}^{k}\right)+f_{\mathrm{DC}}\left(t|\theta_{\mathrm{DC}}^{k}\right)\right)∥}^{2},$$

where $\theta^{k}={\left[\theta_{\mathrm{AC}}^{k},\theta_{\mathrm{DC}}^{k}\right]}^{⊤}$ is obtained via non-linear least-squares. Once estimated, the parameter vectors serve as compact, interpreatable representations (features) of each pulse, enabling subsequent predictive modeling.

To identify the most suitable representation of the AC component, several formulations for $f_{\mathrm{AC}}\left(t|\theta_{\mathrm{AC}}^{k}\right))$ have been proposed in the literature [20,21,54]. In this work, nine of these models were evaluated, constructed from three types of basis functions—Gaussian (see Equation (6)), polynomial (see Equation (7)), and Fourier series (see Equation (8)). The resulting vector of coefficients or parameters of each fit was then considered as features $\theta_{\mathrm{AC}}^{k}$ of the pulse.

Equation (6) shows the summation of $n_{G}$ Gaussians over time (*t*), where $a_{i}$ is the amplitude, $\mu_{i}$ is the mean, $\sigma_{i}$ is the standard deviation of the curve, and *b* is an offset value:

(6) $$f_{\mathrm{AC}}^{G}\left(t|\theta_{\mathrm{AC}}\right)=\sum_{i=1}^{n_{G}}\left(a_{i}\cdot \exp\left(-\frac{{\left(t-\mu_{i}\right)}^{2}}{2\sigma_{i}^{2}}\right)\right)+b,$$

with $n_{G}=\left\{2,3,4\right\}$ having been considered.

Equation (7) is the polynomial expansion to the $n_{P}$-th degree:

(7) $$f_{\mathrm{AC}}^{P}\left(t|\theta_{\mathrm{AC}}\right)=a_{n_{P}}\cdot t^{n_{P}}+a_{n_{P}-1}\cdot t^{n_{P}-1}+\cdots +a_{1}\cdot t+a_{0},$$

with $n_{P}=\left\{6,7,8\right\}$ having been considered.

Equation (8) describes the generic Fourier series expansion to the $n_{F}$-th order, where ω is the angular frequency:

(8) $$f_{\mathrm{AC}}^{F}\left(t|\theta_{\mathrm{AC}}\right)=a_{0}+\sum_{i=1}^{n_{F}}\left(a_{i}\cdot \cos\left(i\cdot \omega t\right)+b_{i}\cdot \sin\left(i\cdot \omega t\right)\right),$$

with $n_{F}=\left\{2,3,4\right\}$ having been considered.

Details of the implemented models and their parameters are given in Table 1.

The fits of the model curves to the PPG pulse waveform were evaluated using the $R_{\mathrm{adj}}^{2}$ score from Equation (9), where $R^{2}$ is the traditional goodness of fit score (see Equation (10)), $l_{s}$ is the number of samples in the curve and $l_{p}$ is the number of parameters in the model, calculated as the sum of linear ($l_{p-l}$) and non-linear ($l_{p-n}$) parameters:

(9) $$R_{\mathrm{adj}}^{2}=1-\left(1-R^{2}\right)\cdot \frac{\left(l_{s}-1\right)}{\left(l_{s}-l_{p}-1\right)},$$

with

(10) $$R^{2}=1-\frac{SS_{\mathrm{res}}}{SS_{\mathrm{tot}}},$$

where $SS_{\mathrm{res}}$ is the sum-of-squares or residual error from the model, and $SS_{\mathrm{tot}}$ is the sum-of-squares from the fit with a horizontal line.

The frequency parameter ω in the Fourier series expansion had a significant impact on the waveform’s shape, with even small variations in ω causing substantial distortion. To mitigate the error introduced by predicting ω and minimize its effect on the reconstructed waveform, ω was redefined as $\omega =\frac{2\pi }{l_{s}}$, where $l_{s}$ represents the number of samples in the pulse. Instead of using ω as a model parameter, $l_{s}$ was used as a feature in the Fourier series expansion. This approach not only addressed the issue of ω prediction but also provided additional information about the pulse width, which proved valuable for accurate reconstruction. Therefore, Equation (13) redefines the AC waveform for the Fourier series expansion model from Equation (8) based on $l_{s}$, with $n_{F}=4$ for the fourth order:

(11) $$f_{\mathrm{AC}}\left(t|\theta_{\mathrm{AC}}^{k}\right)=a_{0}+\sum_{i=1}^{n_{F}}\left(a_{i}\cdot \cos\left(\frac{2\pi }{l_{s}}\cdot t\right)+b_{i}\cdot \sin\left(\frac{2\pi }{l_{s}}\cdot t\right)\right).$$

#### 2.4.2. Dataset Preparation

For each subject, the pulse sequence was divided chronologically into 70% for model development (training and ten-fold expanding cross-validation) and 30% for final testing. The dataset were split ensuring no major imbalance in the number of hypoxia and normoxia pulses, and excluding the outliers. The sequential dataset was transformed, and features from the four most recent PPG periods or pulses (k=4) were incorporated to ensure that temporal patterns and dependencies within the data were captured effectively for the machine learning pulse prediction models.

The splits were made to ensure no major imbalance in the number of hypoxia and normoxia pulses in the training data while sticking to the percentage split. The outliers are marked as values that exceed three standard deviations from the mean of the combined train and validation set for each individual or subject in each channel. The outliers in the test set are marked using the same mean and standard deviation values as from the combined train and validation set. This was done to prevent data leakage of the test set into the model training and to ensure that the test data resembled the training data as closely as possible.

The sequential dataset was transformed into a tabular format to facilitate its use in machine learning algorithms. In this format, each row represents an instance with clearly defined input features and corresponding target variables. To model temporal dependencies, the dataset was shifted one step forward so that the features from the previous pulse served as inputs to predict the target variable of the current instance. Additionally, to create lag features for the *k*-th step, the data was shifted backward by *k* steps, allowing the inclusion of historical information up to *k* pulses prior to the current instance. This approach ensured that temporal patterns and dependencies within the data were captured effectively for the machine learning models.

In this study, the decision to incorporate features from the four most recent PPG periods (k=4) was guided by empirical observation rather than direct optimization. This choice was made to strike a practical balance between capturing sufficient temporal dynamics and maintaining computational efficiency. Including four consecutive periods allowed the algorithm to effectively model changes in the DC level during oxygen desaturation and restoration events, as these processes typically unfold over multiple cardiac cycles. Moreover, the computational burden of processing a larger number of lagged periods was avoided, ensuring the method remained suitable for real-time applications. While this choice was particularly effective for SpO2 estimation, the optimal number of lagged periods may differ for other physiological parameters, such as venous oxygen saturation [55], heart rate variability [56], or respiratory rate [57] due to respiratory sinus arrhythmia, which may necessitate adjustments.

### 2.5. Predictive Modeling

**Figure 2:**
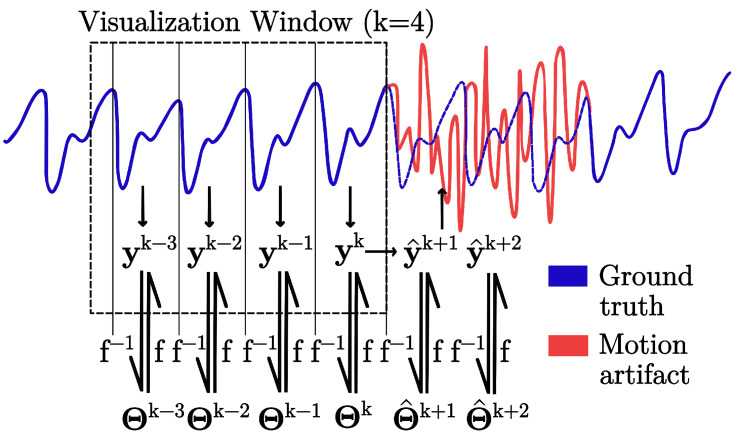
Waveform modeling and forecasting schematic. Four clean pulses of the PPG signal are employed to make predictions of the coming pulses based on a trained model.

The best-performing model from Section 2.4.1 (see Section 3.1) was employed for all the following steps. Figure 2 shows the schematic of the modeling—explained in detail in Section 2.4—and the following forecasting pursued in this work for theoretical pulses affected by motion artifacts.

The components of the PPG model described in Equation (3) can be treated similarly to the standard time-series decomposition framework, which separates data into seasonal, trend, and residual components [58]. Each component (AC and DC) can be forecasted independently and subsequently combined to reconstruct the original signal, using $\theta_{\mathrm{AC}}^{k}$ and $\theta_{\mathrm{DC}}^{k}$ (see Table 2). This representation of PPG pulses as a concise set of features enables the reconstruction of an entire pulse in a single multi-input multi-output (MIMO) prediction step instead of predicting every sample within a pulse, thereby decreasing both computational overhead and the potential for error propagation.

#### 2.5.1. AC Component

The XGBoost model was employed to predict the AC components of the PPG signals, with the xgboost library used for both training and prediction tasks. During training, the objective function was designed to minimize $R_{\mathrm{adj}}^{2}$, specifically tailored for AC component modeling. The model processed data from both the red and the IR channels. The input to the model consisted of four consecutive PPG pulses, denoted as $\left[\theta_{\mathrm{AC},\theta_{\mathrm{AC}}^{k-2}}^{k-3},\theta_{\mathrm{AC}}^{k-1},\theta_{\mathrm{AC}}^{k}\right]$, to predict the next pulse, ${\hat{\theta }}_{\mathrm{AC}}^{k+1}$, for each subject.

Hyperparameter tuning was performed for the best-performing model using a custom error function,

(12) $$e\left(\theta \right)={\left(f_{\mathrm{AC}}\left(t|\theta_{\mathrm{AC}}^{k}\right)-f_{\mathrm{AC}}\left(t|{\hat{\theta }}_{\mathrm{AC}}^{k}\right)\right)}^{2}+2\cdot |l_{s}-\hat{l_{s}}|,$$

where $f_{\mathrm{AC}}$ represents the model function as defined in Equation (13). The term ${\hat{\theta }}_{\mathrm{AC}}^{k}$ denotes the predicted features, and $\theta_{\mathrm{AC}}^{k}$ corresponds to the true approximated features. The parameter $l_{s}$ refers to the number of samples in the pulse, derived from the fourth-order Fourier series fitting, with $l_{s}$ as the true value and ${\hat{l}}_{s}$ as the predicted value. The second term in the error function refines the accuracy of $l_{s}$’ while acting as a regularization term.

For each subject, model hyperparameters were tuned using an expanding time-series ten-fold cross-validation scheme applied within the 70% training portion. In each iteration, the model was trained on an expanding window of earlier pulses and validated on the subsequent segment to preserve temporal dependencies and prevent information leakage. The mean validation error across folds, denoted e(θ), served as the objective function for minimization. The Tree-structured Parzen Estimator (TPE) sampler from Optuna was used for hyperparameter optimization, involving 100 trials. The optimal hyperparameters were averaged across 12 subjects (subjects 1–12) to derive a generalized hyperparameter set. This simulates a realistic scenario where a limited amount of subjects is used to tune the hyperparameters employed for model training on the field.

The best model configuration was retrained on the full 70% training data and evaluated on the 30% held-out test set of all subjects. Once ${\hat{\theta }}_{\mathrm{AC}}^{k+1}$ was predicted, the reconstructed pulse signal ${\hat{y}}_{\mathrm{AC}}^{k+1}$, was obtained and compared against the real signal $y_{\mathrm{AC}}^{k+1}$ using the $R^{2}$ score. This setup ensured robust evaluation of model performance, highlighting its generalization capability for unseen subjects.

#### 2.5.2. DC Component

Ridge regression was used to predict the DC components of the PPG signals, utilizing the Ridge module from the scikit-learn library for model construction, training, and prediction. A regularization parameter of 3 was chosen to balance overfitting and generalization.

The model input consisted of the DC component of four consecutive pulses or periods, $\left[\theta_{\mathrm{DC}}^{k-3},\theta_{\mathrm{DC}}^{k-2},\theta_{\mathrm{DC}}^{k-1},\theta_{\mathrm{DC}}^{k}\right]$, and the output was the predicted next period, ${\hat{\theta }}_{\mathrm{DC}}^{k+1}$.

The reconstructed pulses derived from the model’s output were evaluated using the mean absolute percentage error (MAPE) metric to assess prediction accuracy.

### 2.6. SpO2 Estimation

The performance of the proposed models was evaluated in two aspects: the reconstruction accuracy of PPG pulses and the accuracy of SpO2 estimation derived from the model-generated signals.

For SpO2 estimation, the R-values were computed independently for both the original PPG signals and the predicted signals according to Equation (1). The agreement between R-values obtained from original (*R*) and reconstructed pulses ($\hat{R}$) was assessed using Bland–Altman analysis, where the LoA were defined as ±1.96·σ, with σ denoting the standard deviation of the differences.

Subsequently, two calibration curves were derived using a weighted linear regression between the reference SpO2 values obtained from the pulse oximeter and the corresponding *R* and $\hat{R}$. Calibration was performed using data from subjects 2–16, as reference SpO2 recordings for subjects 1 and 17 were unavailable due to technical issues (see Equation (2)).

Finally, the RMSE was calculated between the SpO2 values estimated from the original (SpO2) and predicted pulses ($\hat{SpO2}$) to quantify the accuracy of the reconstruction. In addition, the error in R-values was propagated through the calibration curve to infer the expected percentage error in the SpO2 estimation obtained from model-predicted waveforms ($\hat{SpO2}$).

## 3. Results and Discussion

### 3.1. Feature Selection

Table 3 shows the mean adjusted $R_{\mathrm{adj}}^{2}$ score for all fits in Table 1.

**Figure 3:**
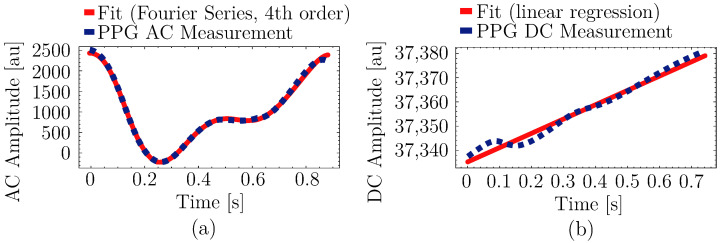
Sample fits of the curve-fitting models for the PPG signal components: (**a**) 4th order Fourier series on an AC pulse, and (**b**) linear fit on a DC segment.

It was observed that the fourth-order Fourier series parameters were the best fit, with $R_{\mathrm{adj}}^{2}$ greater than 0.99, and performed well in capturing the inflection points on the PPG waveform compared to the other fits, as depicted in Figure 3a. The performance of the linear regression for the DC component shown in Figure 3b was also satisfactory.

Therefore, the final AC waveform is given by Equation (13):

(13) $$f_{\mathrm{AC}}\left(t|\theta_{\mathrm{AC}}^{k}\right)=a_{0}+\sum_{i=1}^{4}\left(a_{i}\cdot \cos\left(\frac{2\pi }{l_{s}}t\right)+b_{i}\cdot \sin\left(\frac{2\pi }{l_{s}}t\right)\right).$$

Here, $a_{i}$ and $b_{i}$ are the linear coefficients of the fourth-order Fourier series, and $l_{s}$ is a non-linear coefficient that represents the number of samples in the modeled PPG pulse. Therefore, the AC waveform can be defined with parameters $\theta_{\mathrm{AC}}^{k}={\left[a_{0},a_{1},a_{2},a_{3},a_{4},b_{1},b_{2},b_{3},b_{4},l_{s}\right]}^{T}\in R^{10\times 1}$.

Accordingly, the final DC component can be defined as in Equation (4), with parameters $\theta_{\mathrm{DC}}^{k}={\left[p_{0},p_{1}\right]}^{T}\in R^{2\times 1}$.

It is worth noting that feature selection was not applied in the modeling of the Fourier series. Incorporating this step could potentially enhance the model’s performance by removing irrelevant features or transforming them into more predictive formulations.

### 3.2. Forecasting Performance

#### 3.2.1. AC Forecasting

**Figure 4:**
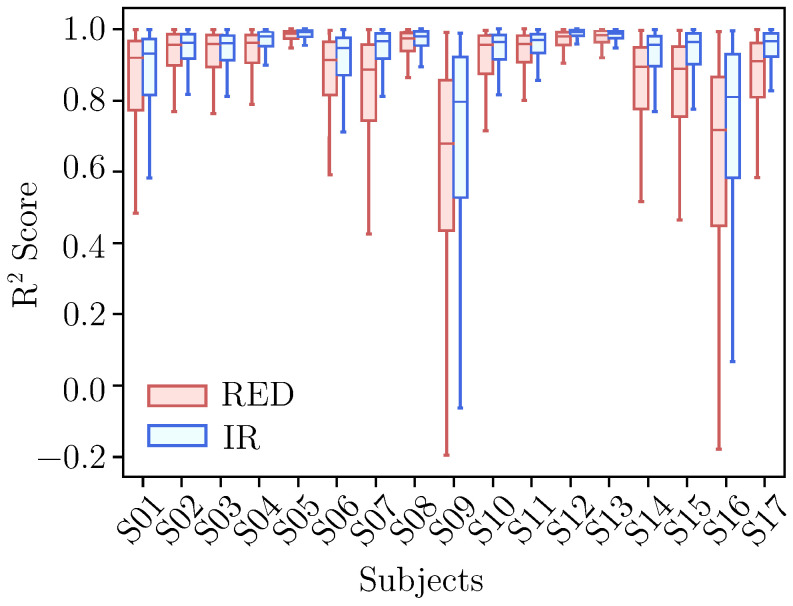
$R^{2}$ scores’ distribution information of predicted AC components ($\theta_{\mathrm{AC}}^{k+1}$) for the red and IR PPG channels.

The final set of hyperparameters, determined as outlined in Section 2.5.1, was as follows: maximum depth = 5, maximum leaves = 8, learning rate = 0.47, regularization parameter (λ) = 0.59, and subsample ratio = 0.74. The results of testing the final set of hyperparameters are presented in Figure 4.

Overall, the predictions performed better for the IR channel compared to the red channel. All seventeen subjects exhibited mean $R^{2}$ scores above 0.9, except for subjects 9 and 16, whose mean $R^{2}$ scores were below 0.8 and were accompanied by significantly broader confidence intervals.

**Figure 5:**
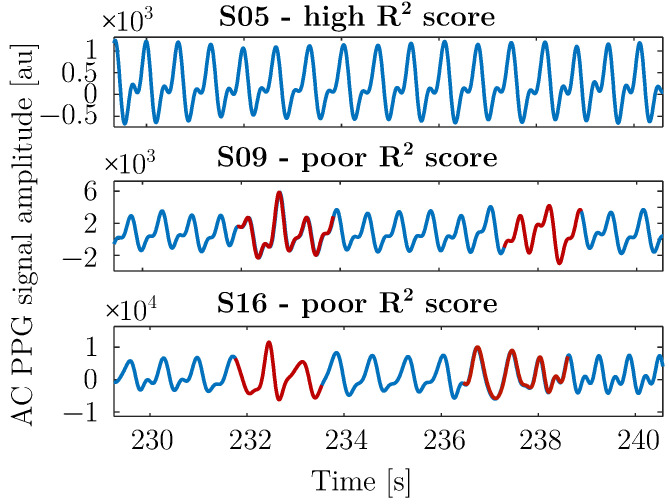
Exemplary AC components of PPG signal extracts of subjects 5 (S05), 9 (S09), and 16 (S16). The motion artifacts, responsible for the poorer $R^{2}$ score of S09 and S16, are highlighted in red.

In an additional analysis conducted to understand the lower $R^{2}$ scores observed for subjects 9 and 16, it was identified that these two subjects experienced substantial contamination from motion artifacts. These artifacts were likely caused by improper sensor placement during the study, which may have compromised the quality of the recorded signals (see Figure 5). The negative values of $R^{2}$ for these two subjects indicate a fit of the model worse than that of a horizontal line, according to Equation (10), which is consistent with the fact that the models were partly trained on corrupted data, which does not match the real physiological waveform of the subject.

The impact of these artifacts is clearly illustrated in Figure 5, where the pulsatile component of the PPG signal for subject 5—who achieved the highest $R^{2}$ score according to Figure 4—shows an excellent signal progression of the AC component with minimal noise that enabled highly accurate predictions. In contrast, subjects 9 and 16 exhibit poor signal quality, with substantial distortion in the pulsatile component, which degraded performance because corrupted pulses were included among the predictor inputs. These cases illustrate the need for a preceding motion artifact detection step to ensure that only clean reference pulses are used for reconstructing corrupted signals.

It is noteworthy that subjects 4 and 17 were female, and subject 15 had a Fitzpatrick skin phototype V (FSPC). Despite the limited number of samples available for each category, the models did not exhibit degraded performance relative to other groups in terms of $R^{2}$ score or MAPE. This observation suggests that the subject-specific training approach contributed to the model’s ability to generalize effectively across individuals.

On a central processing unit (CPU) with 16 GB memory, an Intel i7 microprocessor, and no graphics processing unit (GPU), tuning the hyperparameters for a subject took an average of 4 ± 1 min. The average time taken by the tuned model to train on 1200 pulses was 96 ± 8 milliseconds.

#### 3.2.2. DC Forecasting

**Figure 6:**
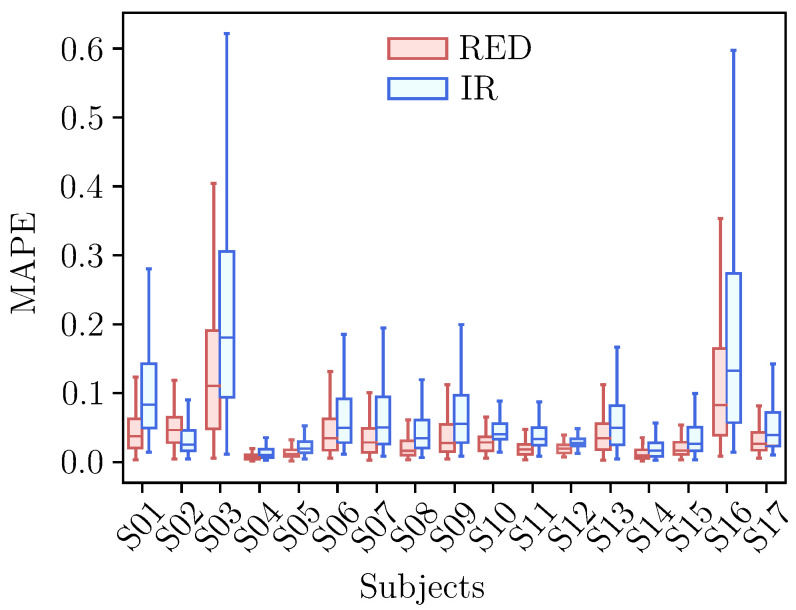
Error distribution information of reconstructed DC components for the red and IR PPG channels: MAPE distribution information of pulses reconstructed from the predicted $\theta_{\mathrm{DC}}^{k+1}$.

The performance of the linear regression model for the DC component is shown in Figure 6.

The reconstruction error was within the desirable range for all subjects with mean MAPE below 0.04, except for subjects 3 and 16. As previously discussed, the motion artifacts affecting subject 16 also had a significant impact on the DC component, resulting in a higher MAPE. Interestingly, the artifacts did not affect the MAPE of subject 9 in the same way. Conversely, the PPG signals for subject 3, which did not exhibit visible motion artifacts in the AC component, showed a notably higher MAPE compared to other subjects. While the exact cause of this anomaly remains unclear, it may indicate a different underlying factor, potentially linked to rapid DC changes during hypoxia events, which could impair the effectiveness of the linear model.

The average time taken to train the model was 2.2 ± 0.4 milliseconds.

#### 3.2.3. Optimal Training Size

Analyzing performance across 10 different training set sizes, each incrementing by one-tenth of the total training sample size, the results from the XGBoost model reported an average $R^{2}$ score of 0.87 ± 0.04. The ridge regression model results presented an average MAPE value of 0.07 ± 0.06 until reaching 50% of the total training size. On further increase, the performance varied due to the possibility of overfitting. Therefore, on average, a maximum of 600 pulses and a minimum of 120 pulses is recommended for model training.

### 3.3. SpO2 Estimation Performance

The calibration curves for the real (original) and predicted PPG pulses were independently estimated as described in Section 2.6. When the regression coefficients were rounded to whole numbers, both calibration curves became identical.

For the ground truth (original) PPG pulses, the calibration curve was given by

(14) $$SpO_{2}=103-15\cdot R,$$

where *R* denotes the R-values computed from the original PPG pulses, and $SpO_{2}$, the corresponding SpO2 levels. Similarly, the calibration curve to estimate the SpO2 levels for the predicted pulses (${\hat{SpO}}_{2}$) was defined using the R-values derived from the predicted PPG pulses ($\hat{R}$).

**Figure 7:**
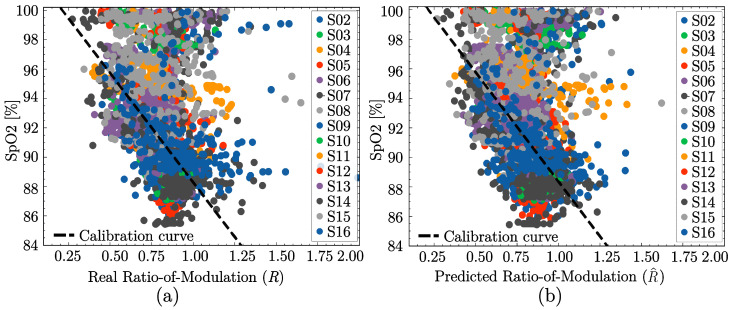
Calibration curves computed manually by weighted linear-fit from (**a**) the recorded data, and the (**b**) predicted data.

These equations are superimposed on the scatter plots from Figure 7, which represent the R-values calculated from the (a) real and (b) predicted PPG pulses in the x-axis and the reference SpO2 values from the reference pulse oximeter in the y-axis. The nearly identical curves prove the effectiveness of the prediction algorithm proposed in this work. It is important to note that the Philips patient monitor used as the reference for SpO2 data averages over 10–15 s of data, resulting in delays compared to instantaneous SpO2 values. Thus, the quality of the scatter plots in Figure 7a,b could be improved. Furthermore, we recall that no motion artifact detection algorithm was applied to the dataset (see Section 2.3), so the data of some subjects were trained with corrupt PPG signal pulses. However, the R-values estimation from the predicted pulses ($\hat{R}$) closely aligns with R-values calculated from the recorded ground truth (*R*).

**Figure 8:**
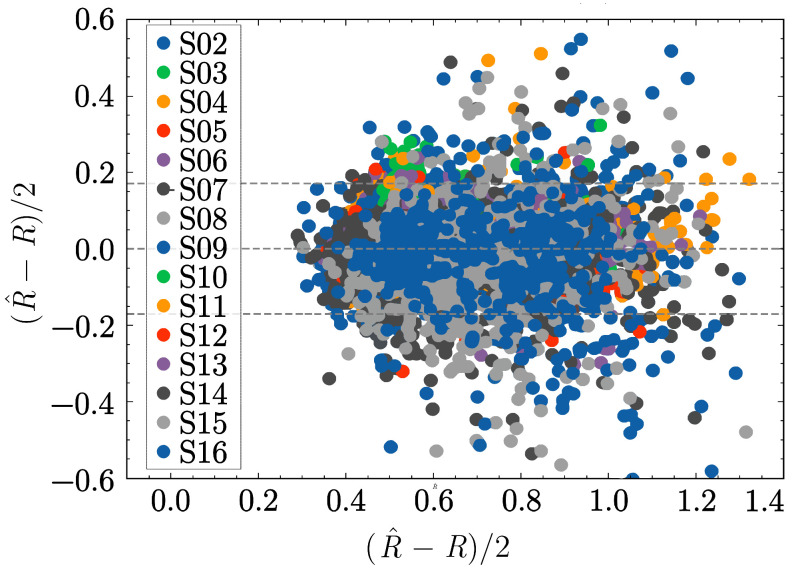
Bland-Altman plot for R-values calculated from the original (*R*) and the predicted ($\hat{R}$) pulses. The dashed lines represent the mean value, and the 95% confidence intervals.

To assess the accuracy of the R-values computed from the predicted values, a Bland-Altman plot was generated, which illustrates the agreement between the real and predicted R-values (see Figure 8). The forecasted outputs yielded R-values with an error of ±0.17 in the 95% confidence interval, suggesting that the reconstructed values achieved satisfactory agreement with the real values.

To benchmark the results and compute the error in the predicted SpO2 values, we used the calibration curve from Equation (14) and considered the slope and the intercepts of original and predicted pulses to be almost equivalent:

(15) $$\begin{array}{c} SpO2-\hat{SpO2}\approx -15\cdot \left(R-\hat{R}\right). \end{array}$$

Substituting the error $R-\hat{R}=\pm 0.17$ into Equation (15), we conclude that the error margin of the SpO2 values estimated from the predicted PPG signals—for subjects 2 to 16 in the saturation range between 85% and 100%—falls within ±2.5% with 95% confidence. The RMSE between the SpO2 values estimated from the predicted signals and the reference values was 1.28% over the entire range.

## 4. Conclusions

This study presents a novel approach capable of real-time predictive modeling of PPG signals corrupted by motion artifacts, enabling accurate SpO2 estimation. A key advantage of the proposed method is its subject-specific training approach, which leverages previous clean segments of data to build lightweight, fast, and efficient models, simplifying hardware implementation. By exploiting short segments of clean data to forecast subsequent pulses, the method adapts to each subject’s unique PPG morphology, which varies with factors such as physiology, ethnicity, and gender. Unlike pooled-data approaches, this individualized modeling reduces dependency on external sensors and heavy training datasets. The framework operates within milliseconds per pulse, ensuring real-time applicability, and achieves good accuracy across the hypoxia range of 85–100%, with an SpO2 RMSE of 1.28% derived from the predicted pulses.

Overall, the findings demonstrate a proof-of-concept for real-time, subject-specific prediction of PPG signals under hypoxia. The results show that accurate SpO2 estimation from model-predicted pulses is technically feasible and could serve as the basis for future artifact-resilient monitoring systems if combined with an artifact detection algorithm. While the results are promising, the approach should still be regarded as a proof-of-concept rather than a clinically deployable solution at this stage.

Although the dataset was limited to healthy young volunteers (including two females and five different skin tones according to FSPC), the algorithm nonetheless showed consistently strong performance across this demographic variability. Due to ethical and logistical constraints, data collection was conducted internally with institute staff, which limited the number of available female participants. Future studies should therefore include a broader cohort encompassing more female participants, a higher number of darker skin tones, older age groups, and varying body mass indices (BMI) to further evaluate the generalizability of the proposed approach. Another interesting direction for future work will be to examine individuals with circulatory or other health conditions, as these factors are likely to influence PPG waveform characteristics relevant to SpO2 estimation more strongly than gender or skin tone. Ethical constraints prevented inclusion of such subjects in the present study, since all participants were required to pass a cardiac stress test prior to controlled hypoxia.

**Figure 9:**
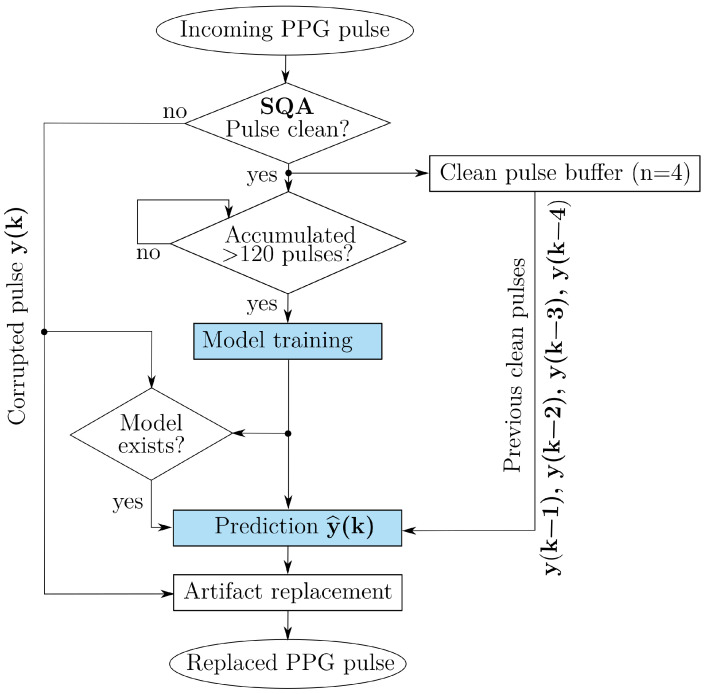
Block diagram of the working principle of the predictive approach in an ideal deployment. The blue boxes represent the algorithm introduced in this work.

If validated in broader and more diverse settings, the algorithm could be embedded into a fitness tracker or a wearable with predetermined hyperparameters. Clean PPG segments would be used to train the subject-specific model for every patient. The model would then make predictions for pulses affected by motion artifacts previously identified with established real-time signal quality assessment (SQA) algorithms. Figure 9 illustrates the working principle of the algorithm in an ideal real-time deployment scenario. The algorithm is envisioned to operate in conjunction with an SQA module, which would detect corrupted pulses and replace them with model-based predictions generated from the preceding four clean signals. This framework differs conceptually from prior approaches that focus on correcting or filtering motion artifacts (e.g., through empirical mode decomposition or adaptive denoising). Instead, our method would substitute artifact-contaminated pulses with physiologically consistent, model-derived predictions based on subject-specific dynamics.

Furthermore, because the proposed framework predicts the AC and DC component of each PPG pulse in real time, future work could validate its utility to estimate additional vital signs, including heart rate (HR), heart rate variability (HRV) and respiratory rate (RR). In particular, beat-to-beat timing (for HR/PRV) and respiratory-driven intensity/baseline modulations (for RR) could be validated under hypoxia and motion.

This work introduces two paradigm shifts in non-invasive monitoring with PPG: moving from large, population-trained models to lightweight approaches that emphasize subject-specific adaptation and performance, and shifting from post hoc correction of MA to predictive modeling that anticipates and manages their impact on the signal. This philosophy addresses longstanding challenges of inter-subject variability while enabling efficient hardware integration for real-time deployment. Our findings demonstrate that artifact-resilient SpO2 estimation can thus be achieved with robustness, computational efficiency, and high accuracy across normoxic and hypoxic events. These results highlight a practical path toward scalable, personalized, and reliable physiological monitoring for wearable scenarios.

## Figures and Tables

**Table 1:** Model fits implemented and compared for the AC waveform: type, order, linear and non-linear parameters ($l_{s}$), and number of parameters to fit ($l_{p}=l_{p-l}+l_{p-n}$).

Model	Order	Linear *P* ($l_{p-l}$)	Non-Linear *P* ($l_{p-n}$)
Sum of Gaussians	2	$a_{1},a_{2},b$ (3)	$\sigma_{1,2},\mu_{1,2}$ (4)
	3	$a_{1-3},b$ (4)	$\sigma_{1-3},\mu_{1-3}$ (6)
	4	$a_{1-4},b$ (5)	$\sigma_{1-4},\mu_{1-4}$ (8)
Polynomial Expansion	6	$a_{0-6}$ (7)	-
	7	$a_{0-7}$ (8)	-
	8	$a_{0-8}$ (9)	-
Fourier Series Expansion	2	$a_{0},a_{1-2},b_{1-2}$ (5)	ω (1)
	3	$a_{0},a_{1-3},b_{1-3}$ (7)	ω (1)
	4	$a_{0},a_{1-4},b_{1-4}$ (9)	ω (1)

**Table 2:** Summary of prediction models for AC and DC components.

Signal Component	Model	Input	Output
AC	XGBoost	$\left[\theta_{\mathrm{AC}}^{k-3},\theta_{\mathrm{AC}}^{k-2},\theta_{\mathrm{AC}}^{k-1},\theta_{\mathrm{AC}}^{k}\right]$	${\hat{\theta }}_{\mathrm{AC}}^{k+1}$
DC	Ridge	$\left[\theta_{\mathrm{DC}}^{k-3},\theta_{\mathrm{DC}}^{k-2},\theta_{\mathrm{DC}}^{k-1},\theta_{\mathrm{DC}}^{k}\right]$	${\hat{\theta }}_{\mathrm{DC}}^{k+1}$

**Table 3:** Mean adjusted $R^{2}$ values for the different fits.

Model	Mean $R_{\mathrm{adj}}^{2}$
Two Gaussians	0.924
Three Gaussians	0.919
Four Gaussians	0.886
2nd order Fourier	0.973
3rd order Fourier	0.984
**4th order Fourier**	**0.993**
6th degree Polynomial	0.981
7th degree Polynomial	0.985
8th degree Polynomial	0.989

## Data Availability

Data may be made available by the corresponding author upon reasonable request.

## Abbreviations

The following abbreviations are used in this manuscript:

AC	Alternating Current
BGA	Blood Gas Analysis
BMI	Body Mass Index
CI	Confidence Intervals
CPU	Central Processing Unit
DC	Direct Current
FDA	U.S. Food and Drug Administration
FIR	Finite Impulse Response
FSPC	Fitzpatrick Skin Phototype Classification
GAN	Generative Adversarial Network
GPU	Graphics Processing Unit
HR	Heart Rate
HRV	Heart Rate Variability
ICA	Independent Component Analysis
IIR	Infinite Impulse Response
IR	Infrared
LoA	Limits-of-agreement
MA	Motion Artifacts
MAPE	Mean Absolute Percentage Error
MIMO	Multi-Input Multi-Output
PPG	Photoplethysmography
RMSE	Root Mean Squared Error
RR	Respiratory Rate
SaO2	Arterial Oxygen Saturation
SpO2	Peripheral arterial oxygen saturation
SQA	Signal Quality Assessment
XGBoost	Extreme Gradient Boost
